# Exploring the Relationship Between the Acceptability of an Internet-Based Intervention for Depression in Primary Care and Clinical Outcomes: Secondary Analysis of a Randomized Controlled Trial

**DOI:** 10.3389/fpsyt.2019.00325

**Published:** 2019-05-10

**Authors:** Adriana Mira, Carla Soler, Marta Alda, Rosa Baños, Diana Castilla, Adoración Castro, Javier García-Campayo, Azucena García-Palacios, Margalida Gili, Mariena Hurtado, Fermín Mayoral, Jesús Montero-Marín, Crisitina Botella

**Affiliations:** ^1^Department of Basic and Clinical Psychology, and Psychobiology, Universitat Jaume I, Castellón, Spain; ^2^Department of Psychology and Sociology, Universidad de Zaragoza, Zaragoza, Spain; ^3^Aragon Institute for Health Research (IIS Aragon), Zaragoza, Spain; ^4^University Hospital Miguel Servet, Zaragoza, Spain; ^5^CIBER de Fisiopatología de la Obesidad y Nutrición (CIBEROBN), Madrid, Spain; ^6^Department of Personality, Evaluation and Psychological Treatment, Universitat de València, Valencia, Spain; ^7^Department of Psychology, Universitat de les Illes Balears, Palma de Mallorca, Spain; ^8^Primary Care Prevention and Health Promotion Research Network, RedIAPP, ISCIII, Madrid, Spain; ^9^Mental Health Clinical Management Unit, Institute of Biomedical Research of Malaga (IBIMA), Regional University Hospital Carlos Haya, University of Malaga, Málaga, Spain

**Keywords:** depression, Internet-based intervention, acceptability, primary care, expectations, satisfaction

## Abstract

**Background:** Depression is one of the most prevalent psychological disorders worldwide. Although psychotherapy for depression is effective, there are barriers to its implementation in primary care in Spain. The use of the Internet has been shown to be a feasible solution. However, the acceptability of Internet-based interventions has not been studied sufficiently.

**Objective:** To assess the acceptability of an Internet-based intervention (IBI) for depression in primary care, and explore the relationship between expectations and satisfaction and the improvement in the clinical variables in primary care patients receiving this intervention. Furthermore, it offers data about the effects of some sociodemographic characteristics on these acceptability variables and analyzes whether the expectations are related to finalizing the intervention.

**Methods:** Data were based on depressive patients who were participants in a randomized controlled trial. In the present study, we present the data from all the participants in the Internet intervention groups (*N* = 198). All the participants filled out the expectation and satisfaction scales (six-item scales regarding treatment logic, satisfaction, recommending, usefulness for other disorders, usefulness for the patient, and unpleasantness), the Beck Depression Inventory-II, and the secondary outcome measures: depression and anxiety impairment, and positive and negative affect.

**Results:** Results showed that participants’ expectations and satisfaction with the program were both high and differences in expectations and satisfaction depended on some sociodemographic variables (age: older people have higher expectations; sex: women have greater satisfaction). A positive relationship between these variables and intervention efficacy was found: expectations related to “usefulness for the patient” were a statistically related predictor to the results on the BDI-II (*Beta* = 0.364), and the perception of how logical the treatment is (*Beta* = 0.528) was associated with change in the clinical variable. Furthermore, the higher the expectations, the higher the improvements exhibited by the patients in all measures evaluated during the ten intervention modules. High expectations were also directly related to finalizing the intervention.

**Conclusions:** This is the first study in Spain to address this issue in the field of IBIs for depression in primary care. The IBI showed high acceptance related to the intervention’s efficacy and completion. Research on IBI acceptability could help to implement the treatment offered.

Clinical Trial Registration: www.ClinicalTrials.gov, identifier NCT01611818.

## Background

Depression is one of the most disabling and prevalent psychological disorders worldwide (1, 2). It has high personal, social, and economic costs (3, 4), and it is among the most common reasons for consulting a general practitioner (GP) (5, 6). In Spain, depression is the most pervasive mental disorder, with a 12-month prevalence estimated at 3.9% and a lifetime prevalence estimated at 10.5% (2). Furthermore, according to WHO data, in Spain, there is a median of 1 clinical psychologist per 100,000 inhabitants, which is much lower than the European median of 3 psychologists (7). In primary care, it is common to prescribe medication for depressive patients (8), but many patients would also like to receive psychological treatment (9). In addition, psychotherapy for depression has been found to be effective in primary care contexts (5, 10, 11). However, there are barriers to the implementation of psychotherapy in primary care, such as the face-to-face time required, the cost, and the lack of trained professionals, which makes it difficult to reach everyone in need and provide the suitable treatment (12–15). Furthermore, some patients reject the use of traditional mental health services (15) because of stigmatization processes (16). According to previous studies (13, 14, 17), it is necessary to explore the usefulness of other alternatives for delivering psychotherapy.

The use of the Internet has been shown to be a feasible solution for the accessibility problem (18, 19). A growing body of research supports the efficacy of Internet-based interventions (IBIs) for the treatment of depression (17, 20–24). IBIs are self-applied, interactive, multimedia (images, video, etc.) interventions, and they are based on the most effective classic cognitive behavioral therapy (CBT) (25). It is true that in northern Europe (i.e., Holland, Sweden), research related to IBIs is more advanced than in the South, e.g., in Spain, where the use of information and communication technologies (ICTs) by psychologists is lower (22, 26, 27). A recent study in Spain shows that only 26% of the sample of psychologists analyzed used ICTs, and only 2.31% of the sample used web-based interventions (28). The main drawbacks emphasized in this study are potential difficulties related to the therapeutic alliance and the limitations of nonverbal communication, followed by confidentiality issues and technical problems in handling the data (28). To date, only one randomized controlled trial (RCT) in Spain has demonstrated the efficacy of an IBI for depression in primary care (29). This study opens the door for its implementation in natural contexts such as primary care, thus helping to reach anyone in need. As is pointed out above, in Spain, the median of clinical psychologists per inhabitant is much lower than the European median (7).

This sort of Internet-based program for depressed patients could be an appropriate solution in mental healthcare, specifically in primary care (30–33). In fact, this line of research is growing at an impressive rate (34), but little is known about the acceptability of the IBIs in this area (35, 36). A recent study in a primary care setting showed that patients’ acceptance of Internet-based treatments for depression in primary care was low, but it could be increased significantly using a brief intervention to facilitate acceptance using an informational video (35). However, it is important to remark that this study investigated behavioral intention to use an IBI, not acceptability in the sense of satisfaction (35).

Although clinical effectiveness is important, an additional criterion that is likely to affect implementation is the acceptability (37). Acceptability refers to the degree to which patients are satisfied with a service, think it is easy to use, and are willing to use it (38, 39). A treatment is acceptable when it is perceived as appropriate, fair, reasonable, and nonintrusive in addressing a problem (29). Taking an intervention’s acceptability into account, it is possible to improve the adherence and outcomes (40, 41). Some variables related to treatment acceptability are expectations and satisfaction (42–45). The literature shows that positive expectations have been associated with better outcomes (46, 47). Moreover, “satisfaction” provides information about the feasibility of the intervention, helping to optimize its effectiveness (45). There are two models that have been established about the predictions of eHealth acceptance: the technology acceptance model (TAM) model and theunified theory of acceptance and use of technology (UTAUT) model (48, 49). However, research points out the complex nature of acceptance and its determinants (50, 51).

In any case, the importance of treatment acceptability is known, and few studies have focused on its assessment in terms of IBIs and its relationship with effectiveness (29, 35, 42, 43, 45, 52). If the objective is to develop and deploy these kinds of programs in a specific context, we have to be sensitive to different participant attitudes that can increase acceptance (28). Only one study in Spain explores patients’ expectations about an IBI for depression in primary care (32) through a qualitative research design. The results showed good general acceptance of the IBI, detecting possible advantages and suggested improvements, such as the need for individualized and personalized interaction (32). To the best of our knowledge, no study in Spain has examined participants’ expectations and satisfaction with an IBI delivered in primary care, and how these variables are related to sociodemographic variables, clinical outcomes, and dropout rates.

The objectives of the present study are: a) to analyze participants’ expectations and satisfaction with an IBI for depression in primary care in Spain and offer data about the effects of some sociodemographic characteristics on these variables; b) to analyze whether the expectations are related to finalizing the intervention program; c) to analyze the relationship between expectations and satisfaction and the primary outcome measure (depression) in patients receiving this intervention; and d) to study the relationship of these variables (expectations and satisfaction) with other relevant clinical variables assessed through the intervention program (depression and anxiety, impairment and positive and negative affect). We believe this work is important because Spain is a country with a low presence of IBIs, and the study was conducted in the National Health System in primary care, where many patients do not have a high level of expertise in the use of ICTs.

## Methods

### Design and Setting

The current study is a secondary analysis of a multicenter, three-arm, parallel, RCT conducted with depressive patients recruited in primary care (registered under ClinicalTrials.gov NCT01611818). Patients were randomized to receive an IBI program (Smiling is Fun) for depression with low-intensity therapist-guided (LITG), completely self-guided (CSG), or an improved treatment based on the usual care from their GP improved treatment as usual (iTAU). It is important to mention that participants in LITG did not receive human support by phone or in person. They were offered the opportunity to request support by email (29). Patients could ask the psychotherapists questions or advice *via* email messages, with a maximum of three contacts during the treatment period. Four trained psychotherapists randomly contacted the patients by email to offer help with any difficulties or problems encountered when using the program. Patients could also ask a technician for help to resolve problems of a technical nature. In CSG, there was no contact with any therapist, and only technical questions about the computer program could be posed. Only 17 email contacts were made with 13 participants in the condition with support, which represented only 11.9% of the patients in this group (29). Patients did not make much use of the support offered. Thus, the low number of email contacts requesting assistance from the psychotherapists and, therefore, the results for the supported and unsupported groups were similar in adherence and efficacy. There were no significant differences between the two groups at any time (29). Because no differences were found in the two intervention conditions in the RCT, the analyses in the present work were performed with the entire sample (LITG and CSG). In this study, we present the data on expectations and satisfaction of all the participants in the IBI groups. The research protocol and data collection procedure have been described elsewhere (29, 53).

### Participants

In the original study, a total of 296 depressive patients were recruited in primary care settings between November 2012 and January 2014 in the Spanish regions of Aragón, Andalucía, Baleares, and Valencia. Recruited participants had mild or moderately severe symptoms according to the Spanish Beck Depression Inventory-II (BDI-II) (54) (14–19: mild depression; 20–28: moderate depression). They were 18–65 years old, had the ability to use a computer, had Internet at home and an email account, and were able to read and understand Spanish. The Mini International Neuropsychiatric Interview (MINI) 5.0 (55) was used to assess different psychological disorders and establish the diagnosis. Patients were excluded from the study if they had severe depression (score ≥29 on the BDI-II), had a severe Axis I psychiatric disorder (e.g., psychotic disorders, presence of suicidal ideation or plan, alcohol/substance abuse or dependence, dementia), or were currently receiving or have received psychological treatment in the previous year. For the present study, the 198 participants who completed the Internet-based program were selected.

### Intervention and Protocol Modules

The protocol used for the treatment of depression is a completely self-help (unguided) Internet-based program called “Smiling is Fun,” developed within the framework of the European project OPTIMI: Online Predictive Tools for Intervention in Mental Illness (grant agreement number 248544). This program is based on CBT, including behavioral activation (56, 57), and it combines strategies to promote emotion regulation, coping capacity, and resilience. At the beginning of the program, there are two initial modules (“Home” and “Welcome”). The “Home” module explains the goals of the intervention, who can benefit from it (with case examples), the terms and conditions, and who we are; and the “Welcome” module informs the participant about the contents and benefits of each module. Regarding the therapeutic content, Smiling is Fun is composed of 10 intervention modules with different psychological techniques, as **Table 1** shows. Smiling is Fun has three complementary transversal tools that can be found in the main menu of the intervention program. The participants can use them every day to receive helpful feedback that is important for their reinforcement and motivation: 1) The “activity report” is where participants first have to specify their mood state, coping ability, and stress on a scale from 0 to 10. Then, they have to rate the degree of satisfaction with each activity in the past 24 h, and how much these activities are related to their own goals and values in life. Moreover, participants have to indicate what percentage of the day they have been active or involved in their life. 2) The “calendar” provides participants with information about homework and tasks already completed. Moreover, it indicates the days that participants have completed the activity report. The participants filled out the activity report every time they accessed the program. 3) “How am I?” offers a set of graphs and feedback on the participants’ progress, including activity level, emotional distress (anxiety and sadness), positive emotionality (active, energetic, enthusiastic, etc)., and negative emotionality (angry, stressed, tense, etc). Patients should work on each module in a sequential way for at least a week. For more information about this Internet-based program, see Refs. (29, 43, 53, 58).

**Table 1:** Intervention modules and main objectives.

Intervention modules	Main objective
1. Medication management	To emphasize the importance of medication
2. Sleep hygiene	To teach the main difficulties with sleep and how to manage them
3. Motivation for change	To analyze the patient’s motivation and emphasize the importance of the treatment
4. Understanding emotional problems	To explain to the patient some typical emotional responses to stressful events
5. Learning to move on	To teach the importance of the activity for our well-being and for getting involved in life
6. Learning to be flexible	To learn to be more flexible in our way of thinking in different situations
7. Learning to enjoy	To teach the role of positive emotions and promote involvement in pleasant and significant activities
8. Learning to live	To understand the concept of well-being and the importance of identifying one’s own psychological strengths and life values and goals
9. Living and learning	To develop an action plan to promote one’s psychological strengths
10. From now on, what else?	To fortify what was learned during the program and analyze future goals

### Measures

The study measures and area and time of assessment are summarized in **Table 2**. Because we had already published a paper with all the efficacy data on the Internet-based treatment program (29), in the present work, we focused only on the primary outcome measure (BDI-II) and on the measurements obtained throughout the entire treatment, that is, measures that were assessed after each intervention module. Furthermore, we decided to report the sociodemographic measures that are commonly included in clinical trials: gender, age, marital status, and level of education.

**Table 2:** Study measures, assessment area, and time of assessment.

Outcomes	Concept	Instrument	Pre-T	Post-T	Post-M	Post-M2
Diagnostic interview	Diagnosis	MINI	X			
Primary	Depression	BDI-II	X	X		
Secondary	Positive and negative affect	PANAS			X	X
	Anxiety impairment	OASIS			X	X
	Depression impairment	ODSIS			X	X
Acceptance	Expectation of treatment	Expectation of Treatment Scale				X
	Satisfaction of the Treatment	Satisfaction of Treatment Scale		X		

Pre-T, pretreatment; Post-T, posttreatment; Post-M, postmodule evaluation; Post-M2, postmodule 2 evaluation; MINI, Mini International Neuropsychiatric Interview; BDI-II, Beck Depression Inventory-II; PANAS, Positive and Negative Affect Scale; ODSIS, Overall Depression Severity and Impairment Scale; OASIS, Overall Anxiety Severity and Impairment Scale.

#### Sociodemographic Variables

The following sociodemographic variables were collected: gender, age, relationship status, and level of education.

#### Diagnostic Interview

##### Mini International Neuropsychiatric Interview 5.0 (MINI)

It is a structured diagnostic psychiatric interview based on the *Diagnostic and Statistical Manual of Mental Disorders, Fourth Edition (DSM-IV)* and the *International Classification of Diseases*. The MINI can be administered in a short period of time by (55) a clinician with brief training. In this study, the Spanish validated version was used (59).

#### Primary Outcome Measure (Pre- and Postassessment, Over the Internet)

##### Beck Depression Inventory-II, Spanish Validated Version

The *Beck Depression Inventory-II* (BDI-II), Spanish validated version (60), assesses the presence and severity of depressive symptoms. It is a self-report measure with 21 items rated on a 4-point scale ranging from 0 (not present) to 3 (severe), and total scores can range from 0 to 63. It contains emotional, cognitive, and somatic symptoms of depression, and it is based on the diagnosis of major depressive disorders described in the *Diagnostic and Statistical Manual of Mental Disorders, Fourth Edition (DSM-IV)*. The Spanish version of the BDI-II (60), as a continuous variable, was used as the primary outcome measure. The BDI-II is one of the most widely used instruments to measure depression severity, and the published studies show strong agreement between the BDI-II and the clinical diagnosis of depression, as well as good psychometric properties for the scale (60, 61). In the current study, Cronbach’s alpha reliability coefficient was very large (α = .91).

#### Secondary Outcome Measures (Postmodule Assessment, Over the Internet)

##### Overall Depression Severity and Impairment Scale (ODSIS)

The Overall Depression Severity and Impairment Scale (ODSIS) is a self-report measure with five items that evaluate experiences related to depression (62). It measures the frequency and severity of depression, as well as the level of avoidance, work/school/home interference, and social interference associated with depression. It was found to have good convergent and discriminant validity and excellent internal consistency (α = 0.94 in the outpatient sample, 0.91 in the student sample, and 0.92 in the community sample) (62). The ODSIS was translated into Spanish, and a validation process was carried out (63). The validation data confirmed the factorial structure and reliability and validity data obtained by the original authors (62). In the current study, Cronbach’s alpha reliability coefficient was very large (α = .92).

##### Overall Anxiety Severity and Impairment Scale (OASIS)

The Overall Anxiety Severity and Impairment Scale (OASIS) consists of a five-item questionnaire, rated from 0 to 4, that assesses the frequency and severity of anxiety symptoms (64). The instrument also provides measures of avoidance, as well as work, academic, social, and everyday-life impairment related to anxiety symptoms. A psychometric analysis of the OASIS scale found good internal consistency (α = .80), test–retest reliability (*k* = .82), and convergent validity for this instrument. The Spanish version of the OASIS showed good internal consistency (α = .86), convergent and discriminant validity, as well as sensitivity to change (65). In the current study, the alpha coefficient was very satisfactory (α = .85).

##### Positive and Negative Affect Scale

The *Positive and Negative Affect Scale* (PANAS) consists of 20 items that evaluate two independent dimensions: positive affect (PA) and negative affect (NA). The range for each scale (10 items on each) is from 10 to 50 (66). It is a brief, reliable, and valid self-report measure, and it has shown excellent convergent and divergent validity (54). The Spanish version has demonstrated high internal consistency (α = 0.89 and 0.91 for PA and NA in women, respectively, and α = 0.87 and 0.89 for PA and NA in men, respectively) in college students (67). In the current study, alpha coefficients were very satisfactory for both scales (αs = .92 and .89 for positive and negative subscales, respectively).

#### Treatment Acceptance Measures

##### Expectation and Satisfaction Scales (Expectations at the End of Intervention Module 2 and Satisfaction at Postassessment, Over the Internet)

These scales are based on the Borkovec and Nau questionnaires (68). Each scale has six items, with responses ranging from 0 (“not at all”) to 10 (“very much”). The maximum of the scores in both scales is 60. More punctuation means more expectation and satisfaction. The questions assess how logical the intervention seemed, to what extent it satisfied the patient, whether the intervention would be recommended to other patients, whether it would be useful to treat other problems, its usefulness for the patient’s specific problem, and to what extent it could be unpleasant. Item 6, “unpleasantness,” is answered in reverse. Patients completed the expectation scale at the end of the second module, once the intervention rationale had been explained. The satisfaction scale was completed at the end of the program. These scales have been used in several studies (42–44, 69, 70). In the current study, Cronbach’s alpha reliability coefficients were very satisfactory for both scales (αs = .88 and .87 for expectation and satisfaction scales, respectively).

### Procedure

Between November 2012 and January 2014, patients were recruited in primary care settings. General practitioners from the Spanish regions of Andalusia, Aragon, Valencia, and the Balearic Islands detected possible participants using a brief questionnaire. After a few days, an independent researcher used the MINI and other questionnaires to assess the participants, taking into account inclusion and exclusion criteria. Later, participants signed written informed consent to be part of the study. Then, a randomization was carried out by another independent researcher. The ethics committee of the regional health authority accepted the study on April 7, 2010 (ref: PI10/01083). All participants completed the pretreatment evaluation integrated in the web system. When they finished each of the10 treatment modules, they performed the postmodule evaluation, also through the web system. At the end of treatment, they also completed the posttreatment evaluation through the web site. More details about the design, procedure, therapists, and recruitment methods are included in the main outcome study (29).

### Statistical Analysis

Means (M) and standard deviations (SD) for the expectation and satisfaction measures were analyzed. Pearson correlation coefficients, *t*-tests, and one-way ANOVAs were conducted to find out the effect of some sociodemographic variables on the expectation and satisfaction measures. *T*-tests were performed comparing expectations between dropouts and completers. For two-group comparisons, effect sizes were estimated by means of the standardized mean difference (*d*) and interpreted as low, moderate, and large magnitude for *d* values of 0.2, 0.5, and 0.8, respectively (71). For ANOVAs, the effect size was approached with the proportion of variance accounted for *Eta*^2^. Moreover, multiple regression analyses (by means of stepwise and hierarchical models) were applied to examine whether expectations and satisfaction predicted the improvement in the primary outcome measure. The improvement was defined as the difference between the pretest and posttest scores in the BDI-II. Finally, a canonical correlation analysis was performed with the six expectation items as the predictor variables and the 10 postmodule evaluations of the secondary outcome measures as the dependent variables. Variables with structure coefficients (*r*_s_) larger than .45 (in absolute value) were considered relevant for interpreting the results of these analyses (72). All the statistical analyses were conducted with IBM startical product and service solutions (SPSS) Statistics 20 (IBM Corporation, Armonk, NY, USA).

## Results

### Sample Description

Final analyses were performed on 198 subjects. The sample was mainly composed of women (76.4%). The age range was between 25 and 69 years, with an M of 48.33 (SD: 9.99). In the case of the educational level, 12.12% had primary studies, 68.8% had secondary studies, and 18.18% had university studies. Regarding marital status, 12.3% were single, 69.2% were married or had a partner, and 16.9% were separated or divorced. Regarding depression severity at pretreatment, the average on the BDI-II was 23.50 with a standard deviation of 8.40. One hundred eight participants were dropouts (54.54% of the sample).

### Participants’ Expectations and Satisfaction

**Table 3** presents the mean (M) and standard deviation (SD) of each item on the expectation and satisfaction scales, as well as their total scores. Note that item 6 (“unpleasantness”) is answered in reverse (the higher the score, the lower the expectation or satisfaction), and so it has been recoded to follow the same scoring criteria as the other five items on the scale. Therefore, taking into account that the scale ranges from 0 to 10 points, the average levels of expectations seem high, as all of them are above or around 7. Moreover, the average levels of satisfaction seem high, as all of them are above 7.5 points. In addition, a positive, statistically significant relationship was found between expectations and satisfaction (*r* = .68, *p* < .001).

**Table 3:** Means and standard deviations for expectation and satisfaction scales.

Statements	Expectation M (SD)	Satisfaction M (SD)
1. Treatment logic	7.14 (2.47)	8.07 (1.71)
2. Treatment satisfaction	6.93 (2.49)	7.87 (1.81)
3. Recommending to others	7.37 (2.67)	8.47 (1.76)
4. Usefulness for other disorders	7.32 (1.91)	7.79 (1.72)
5. Usefulness for the patient	7.18 (2.03)	7.57 (1.90)
6. Unpleasantness*	7.31 (2.38)	7.77 (2.10)
Total	7.42 (1.60)	7.92 (1.43)

*Item 6 is answered in reverse, and so it has been recoded to follow the same scoring criteria as the other five items on the scale.

Number of participants (N) on expectation scale (from 1 to 3 items) = 198, N on expectation scale (from four to six items, and total score) = 187, N on satisfaction scale = 90. M, mean; SD, standard deviation.

### Sociodemographic Characteristics of the Participants Related to the Expectations and Satisfaction With the Program

Regarding the relationship between the patients’ ages and their expectations and satisfaction with the treatment, results showed that the only statistically significant correlations were found between age and the items “treatment logic” (Pearson’s *r* = .217, *p* < .01), “treatment satisfaction” (Pearson’s *r* = .248, *p* < .001), and “recommending to others” (Pearson’s *r* = .209, *p* < .01) from the expectation scale. However, there were no statistically significant correlations between age and satisfaction.

Regarding the relationship between the patients’ sex and their expectations, the analysis revealed that there were no statistically significant differences between men and women on any items, or on the total score for expectations. In the case of satisfaction, there was greater satisfaction in women than in men on the items “usefulness for other disorders” (*t*(86) = 2.59, *p* = .011; *d* = 0.65) and “usefulness for the patient” (*t*(86) = 2.60, *p* = .011; *d* = 0.66), and on the total score (*t*(86) = 2.29, *p* = .024; *d* = 0.58). There was also a greater marginally significant satisfaction in women on the items “treatment logic” (*t*(86) = 1.88, *p* = .063; *d* = 0.47) and “recommending to others” (*t*(86) = 1.94, *p* = .055; *d* = 0.49).

The effect sizes (*ds*) were of medium-high magnitude. Regarding marital status, one-way ANOVAs were applied, with marital status as an independent variable and expectations and satisfaction as dependent variables, in order to verify their possible statistical relationship. There were no statistically significant relationships between civil status and expectation or satisfaction scales. The same thing occurred in the case of the educational level.

### Are the Patients’ Expectations Related to Finalizing the Intervention Program?

This section examines whether the expectations of the patients who finish and do not finish the treatment are similar or if, on the contrary, differences between the two groups can be observed (dropouts and completers). **Table 4** presents the results when comparing the M expectations of both groups. Thus, the results showed that, with the exception of item 6 (“unpleasantness”), statistically significant differences were observed in favor of completers on all the items and on the total score. The effect sizes (*ds*) indicated that these differences were of moderate practical relevance. There were no statistically significant differences between dropouts and completers on the BDI-II at preintervention (*t* (196) = −.47, *p* = .640).

**Table 4:** T-tests comparing expectations of dropouts and completers.

Statements	Dropouts		Completers		t	P	*d*
	Mean	SD	Mean	SD			
1. Treatment logic	6.49	2.88	7.91	1.54	−4.42^a^	<.001	−0.63
2. Treatment satisfaction	6.29	2.85	7.71	1.70	−4.35^a^	<.001	−0.62
3. Recommending to others	6.75	3.15	8.12	1.67	−3.91^a^	<.001	−0.56
4. Usefulness for other disorders	6.99	2.02	7.67	1.72	−2.45	.015	−0.35
5. Usefulness for the patient	6.87	2.19	7.51	1.79	−2.19	.030	−0.31
6. Unpleasantness*	7.33	2.30	7.29	2.48	−0.12	.907	0.02
Total	7.15	1.75	7.70	1.36	−2.37	.019	−0.34

*Item 6 is answered in reverse, and so it has been recoded to follow the same scoring criteria as the other five items on the scale. N for dropouts = 108. N for completers = 90. d, Cohen’s d (standardized mean difference).

a The Satterthwaite correction was applied due to heterogeneous variances.

Regarding adherence, 90 participants completed all the modules (45.45% of the sample), and 108 were dropouts (54.54% of the sample).

### Relation Between Expectations and Satisfaction and the Primary Outcome Measure: Beck Depression Inventory-II

For this purpose, we restricted the sample to the 90 patients in the database with BDI-II data on the pretest and posttest. A first analysis consisted in calculating the Pearson correlation coefficients between expectations and the BDI-II at the pretest, in order to investigate potential relations between expectations and BDI-II severity in the baseline. All correlation coefficients between each expectancy item (and its total score) and the BDI-II at the pretest were statistically nonsignificant, with correlations ranging between *r* = −.09 (*p* = .37) for “usefulness for other disorders” and *r* = −.18 (*p* = .09) for the expectation total score.

A stepwise linear regression was conducted taking the expectation items (and their total score) as predictors and the BDI-II pretest–posttest improvement as the dependent variable. In the set of potential predictors, we also included the BDI-II at the pretest in order to control its potential confounding effect on the expectations–depression improvement relationship. The final model included two predictors: the expectation item 5, “usefulness for the patient” (*Beta* = 0.364, *t*(87) = 3.77, *p* < .001), and the BDI-II at the pretest (*Beta* = 0.325, *t*(87) = 3.37, *p* = .001), with 18.7% of total variance explained (*F*(2, 87) = 11.25, *p* < .001). The remaining potential predictors did not reach statistical significance. Next, a hierarchical regression model by successively entering in the model the BDI-II at the pretest and item 5 revealed that the inclusion of the former explained 7.6% of the variance (*F*(1, 88) = 7.19, *p* = .009), and the inclusion of the latter in the model led to an increase of 13% in the percentage of variance explained (Δ*F*(1, 87) = 14.23, *p* < .001). The direction of the relationship between item 5 and the result on the BDI-II was positive: the higher the score on the expectations reflected in item 5, the greater the pretest–posttest change in the BDI-II (*Beta* = 0.364). It should be noted, however, that the proportion of variance explained by these two predictors was low (*R* ^2^_adj_ = .187), which limits the practical use of this model to predict treatment outcome.

In the case of the relation between satisfaction and treatment efficacy, item 1, “treatment logic” (*Beta* = 0.528, *p* < .001), and the BDI-II at the pretest (*Beta* = 0.339, *t*(87) = 3.90, *p* < .001) were selected to enter in the model, accounting for 33.5% of the variance (*F*(2, 87) = 23.47, *p* < .001). Next, a hierarchical regression by successively entering the BDI-II at the pretest and item 1 revealed an increase of 11.3% in the percentage of variance accounted for by the last variable (Δ*F*(1, 87) = 36.83, *p* < .001). The direction of the relationship between item 1 and the result on the BD-II was positive: the higher the satisfaction score reflected in item 1, the greater the pretest–posttest change in the BDI-II (regression coefficient, *b*_1_ = 2.55) (*Beta* = 0.528).

### Relation Between Expectations and Evolution in the Clinical Variables Assessed Through the Intervention Program (Postmodule Assessment)

#### Depression Impairment

The canonical correlation analysis with the six expectation items as the predictor variables and the 10 depression measures as the dependent variables was statistically significant, χ2(60) = 85.656, *p* = .017, with 63.7% of the variance shared between the two variable sets. Only the first function was considered noteworthy in the context of this study (37.3% of shared variance). **Table 1** in the **Supplementary Material** presents the results. With the exception of occasion 1, all of the depression measures significantly contributed to the canonical variable. In particular, depression on occasions 7, 10, 9, and 8 showed a larger shared variance with the canonical variable (73.8%, 68.4%, 58.8%, and 56.8%, respectively). Regarding the predictor variable set, with the exception of expectation 3 (“recommending to others”), all of them exhibited relevant structure coefficients. In particular, expectation 4 (“usefulness for other disorders”) exhibited the largest shared variance with the canonical variable (47.2%). A negative relationship between the canonical variable of depression and the expectation variable was found, indicating that the greater the expectations, the lower the depression exhibited by the patients on the 10 measurement occasions.

#### Anxiety Impairment

The canonical correlation analysis with the six expectations as the predictor variables and the 10 anxiety measures as the dependent variables reached statistical significance, χ2(60) = 90.055, *p* = .007, with 65.6% of the variance shared between the two variable sets. Only the first function was considered noteworthy in the context of this study (34.3% of shared variance). **Table 2** in the **Supplementary Material** presents the main results of this canonical correlation analysis. The structure coefficients show that, with the exception of occasions 1 and 3, all of the anxiety measures significantly contributed to the canonical variable, with occasions 8, 7, and 10 exhibiting a larger shared variance with the canonical variable (51.8%, 50.1%, and 46.4%, respectively). Regarding the predictor variable set, with the exception of expectation 5 (“usefulness for the patient”), all of them exhibited relevant structure coefficients. In particular, expectations 4 (“usefulness for other disorders”) and 1 (“treatment logic”) exhibited the largest shared variances with the canonical variable (78.7% and 54.2%, respectively). Finally, a negative relationship was found between the canonical variable of anxiety and the expectation variable, indicating that the greater the expectations, the lower the anxiety exhibited by the patients on the 10 measurement occasions.

#### Negative Affect

The canonical correlation analysis with the six expectations as the predictor variables and the 10 measures of NA as the dependent variables did not reach statistical significance, χ2(60) = 72.960, *p* = .122, with 57% shared variance between the two variable sets. Although a statistically insignificant relationship was found between expectations and negative affect, the first canonical function reached a relevant percentage of shared variance between the two canonical variables (28%). Therefore, the first canonical function was considered noteworthy. **Table 3** in the **Supplementary Material** presents the results. Only 3 out of the 10 measures of NA significantly contributed to the canonical variable: those of occasions 9, 10, and 5, with 27.7%, 21.2%, and 20.2% of shared variance with the canonical variable, respectively. Regarding the predictor variable set, only expectation 5 (“usefulness for the patient”) exhibited a relevant structure coefficient (36.4% shared variance with the canonical variable). An inverse relationship between expectations and NA was found, indicating that the greater the expectations, the lower the NA exhibited by the patients during the treatment.

#### Positive Affect

The canonical correlation analysis with the six expectations as the predictor variables and the 10 measures of PA as the dependent variables was also statistically significant, χ2(60) = 83.687, *p* = .023, with 62% of the variance shared between the two variable sets. Only the first function was considered noteworthy in the context of this study (32.9% of shared variance). **Table 4** in the **Supplementary Material** presents all the measures of PA that significantly contributed to the canonical variable. In particular, PA on occasions 8, 10, and 9 exhibited a larger shared variance with the canonical variable (81%, 79.9%, and 62.4%, respectively). Regarding the predictor variable set, all of them exhibited relevant structure coefficients. In particular, expectations 1 (“treatment logic”) and 4 (“usefulness for other disorders”) showed the largest shared variances with the canonical variable (66.6% and 57.9%, respectively). A positive relationship was found between the canonical variable of PA and the expectation variable, indicating that the greater the expectations, the greater the PA exhibited by the patients throughout the 10 measurement occasions.

## Discussion

The first objective of this work was to analyze participants’ expectations and satisfaction with an IBI for depression in primary care in Spain. Furthermore to offer data about the relationships among some sociodemographic characteristics and these variables; and moreover, to analyze whether the expectations were related to finalizing the intervention program. Furthermore, this study offers data on the associations between expectations and satisfaction and the primary outcome measure (depression) in patients receiving this intervention, as well as the relationship between the expectations and the other clinical variables assessed through the intervention program (depression and anxiety impairment and positive and negative affect).

The participants in this study reported positive expectations about the program, coinciding with other studies on Internet-based treatments (22, 43, 73, 74) and also in a primary care setting. The expectations were high, suggesting that participants found the treatment very logical and useful, even for other psychological problems, and they felt confident about recommending it to a friend. Participants’ satisfaction after using the program was also high. Our results coincide with the literature indicating that people treated with Internet-based programs report high levels of acceptability and high satisfaction (58, 73–78). In addition, this finding is consistent with the results obtained in the previous qualitative study, where participants showed a favorable opinion in the case where they had to use an online program (32). However, a recent study carried out in Germany showed that an IBI for depression in primary care settings obtained low acceptance; however, the authors explained that acceptance could be increased by displaying a brief informational video (35). Hence, a possible explanation for the high satisfaction with our IBI can be found in the richness and quality of the ICT support: the program offers information about the treatment and continued personalized feedback to users through various transversal tools for providing support, as described earlier (activity report, calendar, and how am I)?. These results are especially relevant in an ecological environment such as the primary care service in a national health system.

It is also important to mention that the results showed differences in expectations and satisfaction depending on some sociodemographic variables. Regarding the participants’ age, the results showed that the older they were, the higher their expectations (specifically in items of “treatment logic,” “treatment satisfaction,” and “recommending to others”). According to a recent individual patient data meta-analysis (IPDM) (78), younger ages were related to more dropouts. In the present study, older age was associated with better expectations; thus, the results in our study could be related with those found in that study. However, this is only a possible explanation, and it is not possible to have a firm conclusion about this aspect. With regard to the participants’ sex, the analysis showed that there was greater satisfaction in women than in men on the items “usefulness for other disorders” and “usefulness for the patient” and on the total score. The IDPM, mentioned above (78), revealed that men had a greater risk of dropping out. This result could indicate that men are less satisfied than women, as in our study. However, this is only a possible explanation since we don’t have information regarding the satisfaction with the program of the dropout participants. It should also be taken into account that women try harder to cope with depression than men (78), which would indicate a greater willingness to continue and complete IBIs and be more satisfied with them. Moreover, there is also evidence supporting the idea that women are more aware of health problems than men (78, 79). Furthermore, the prevalence data indicate that there are also more depressed women than men (2, 80), which may influence the difference in acceptance. Regarding marital status, the results revealed that there were no statistically significant differences among the different categories on the expectation and satisfaction scales. The same thing occurred in the case of the educational level.

Another important result is that patients who finished the intervention had higher expectations about it. Taking into account the significant dropout rate from IBIs (73, 81–83), this result shows the relevance of working to improve the participants’ expectations as a way to promote program adherence. The sociodemographic variables and the participants’ expectations could be understood as moderators.

The completion rate in the present study was 45.45% of the sample. Thus, the dropout rates in the present study are high (54.4%), but it is important to take into account that the context of the present study is the primary care setting. Thus, the sample had specific characteristics (with a lower educational level than in other contexts: only 18.18% had university studies). A recent IPDM showed that a lower educational level signiﬁcantly increased the risk of dropping out (78). Furthermore, the literature shows that in unguided web-based interventions for depression, compared to guided web-based interventions, average levels of adherence were estimated at 26% in unguided interventions and 72% in guided interventions (22, 78). Dropout rates in Internet-based treatment programs range from 2% to 83%, with a median of 19% and a weighted average of 31%, due to various causes, one of which could be not providing human support (84). In the present paper, patients did not make much use of the support offered (only 11.9%). For this reason, almost all the participants received unguided intervention, which may have influenced the results regarding dropouts.

Regarding the relationship between the expectation and satisfaction measurements and the primary clinical variable (depression), and the relation between expectations and the clinical variables assessed through the intervention program (depression and anxiety impairment and positive and negative affect), the results showed that expectations related to “usefulness for the patient” were a predictor that was statistically related to the results on the BDI-II. Thus, positive expectations were associated with better outcomes on the primary outcome measure. Furthermore, data are presented for the relationship between expectations and improvements in the clinical variables assessed throughout the intervention program. The results for the negative emotionality variables (depression and anxiety impairment and negative affect) showed that the higher the expectations, the lower the depression and anxiety impairment and NA exhibited by the patients during the 10 intervention modules. In the case of positive emotionality, the results showed that the higher the expectations, the greater the PA exhibited by the patients on the 10 measurement occasions. These results agree with the literature (36, 39) and suggest the importance of considering patients’ expectations about treatment as a factor that explains part of the therapeutic efficacy. Therefore, it is important to inform the participants, before the intervention, about why the program will be useful and how it can help them. Thus, it is important to present the IBI in a way that allows patients to obtain adequate and relevant information that can activate positive but realistic expectations about the intervention. Furthermore, in IBIs where the therapist is less visible, it is important to spend time developing strategies (for example, *via* telephone or with manuals) to emphasize the usefulness of the online intervention.

The literature shows that, in online programs without support during the intervention, preceding human contact increases the effectiveness (20); therefore, it would be interesting during this contact to transmit the rationale and the usefulness of the program. In addition, another strategy would be to dedicate the first modules of the program to this topic. In the IBI used in the present study, the “Home” and “Welcome” modules explain the usefulness of the program by showing examples of people who have similar problems. Regarding satisfaction with the IBI, results showed that the higher the satisfaction score reflected on the “treatment logic” item, the greater the improvement on the BDI-II. Thus, the satisfaction of perceiving the intervention as a logical treatment is related to greater benefits in the clinical variable. This is an important result because satisfaction has been found to help to optimize intervention efficacy (10, 45), and the results in the present study show that the same thing might occur with an IBI program for depression in primary care settings. However, it is important to mention that in the absence of a controlled treatment condition, this submission is not about treatment efficacy. The efficacy of the intervention was shown in the RCT carried out (29).

Based on these results, it is important to take into account the benefit of developing interventions not only with effective treatment components but also with aspects that improve patients’ expectations and satisfaction with the intervention, because the treatments’ completion and effectiveness will improve with the acceptability of the intervention (19, 69, 83). As we pointed out in the introduction, although clinical effectiveness is important, an additional criterion that is likely to affect implementation is the acceptability (37). At this moment, we are working on an implementation study to try to introduce this type of intervention in the National Health System in Spain. The task is not at all simple, given that in primary care in Spain, there are no clinical psychologists. There are only clinical psychologists in specialized care. For psychological treatment, the primary care physician has to refer the patient to specialized care. One of the biggest barriers is clinical professionals’ resistance to adopting this type of program applied through the Internet. Therefore, it is essential to demonstrate with data that this type of treatment works (as seen in the RCT) at an affordable cost. We worked on this cost–benefit issue in another study (85). Furthermore, future research should study therapists’ attitudes toward IBIs in the primary care context to find out how to improve their expectations and satisfaction (24, 86).

## Limitations

The present study has some limitations that should be taken into account. First, the sample size is small, and the dropout rates are high (54.4%). It is true that the real sample size is modest, which is reflected in the limitations and the difficulty of generalizing the results. However, the results are from the first RCT performed in Spain on an IBI for depression. For this reason, although the sample is modest, we think the results are important for future implementations of IBIs in our country or in other Spanish-speaking countries with a Latin culture. Second, the assessments were carried out through the online program. Previous studies indicate that if assessments are carried out through the web, psychometric properties may change (47). However, other studies have demonstrated the usefulness of Internet and telephone assessments, and their concordance with traditional face-to-face assessment (87–90). Third, the effect of the GPs or psychotherapists may be a source of variability, and in the current study, this was not taken into account (29). Fourth, the generalizability of this work is limited by evaluating the participants’ satisfaction with one intervention. Fifth, because we do not have data about the dropouts’ satisfaction with the intervention, we do not know whether satisfaction or other variables moderated the completion of the program. However, following the literature, there might be some reasons that affect the dropout rates, such as the amount of support (insufficient) and the lack of specificity of the intervention contents to the participants’ own psychological problems (91). Sixth, using individual items from the expectation and satisfaction questionnaires limited the psychometric properties of these variables included in our analyses. However, in the balance between psychometric validity and specificity of the information offered by the individual items, we considered it more useful and informative to analyze individual items. Another limitation of our study was the low percentage of variance explained by the multiple regression for expectations on treatment efficacy (18.7% only). This finding limits the usefulness of the predictive model for practical purposes. Finally, the acceptability assessment (expectations and satisfaction) was based on two questionnaires, rather than qualitative information that can provide a general impression of the program. Furthermore, it would have been interesting to have used other assessment measures of acceptance [i.e., the Client Satisfaction Questionnaire adapted to IBIs (CSQ-I)] (92). In addition, the regression models elaborated to find predictors of treatment efficacy were based on the individual items of the expectation and satisfaction scales, not on composite scores, so that the reliability of these indicators was of limited scope. In the future, it would be appropriate to include qualitative analyses to report complementary and detailed data on this topic. Furthermore, it would be interesting to compare the results obtained in the present work with previous studies that we have carried out using the expectation and satisfaction scales.

## Conclusions

Despite its limitations, to the best of our knowledge, this is the first study in Spain to address this issue in the field of IBIs for depression in primary care. The IBI showed high acceptance (expectations and satisfaction) that is related to the intervention’s efficacy. Research on IBI acceptability in relation to completion and efficacy variables could help to implement the treatment offered, reaching more people and improving the outcome. Future research in this field is needed.

## Ethics Statement

We confirm that any aspect of the work covered in this manuscript that has involved human patients has been conducted with the ethical approval of all relevant bodies and that such approvals are acknowledged within the manuscript. The study was approved by the ethics committee of the regional health authority on April 7, 2010 (ref: PI10/01083). All participants interested in participating signed an informed consent form.

## Author Contributions

AM and CS drafted the manuscript, with important contributions from CB, AG-P, and RB. CB, in collaboration with JG-C, MG, FM, and JM-M, designed the study and participated in each of its phases. AM, CS, MA, AC, and MH collaborated in the manuscript development and participated in each study phase. AM, CS, CB, and DC carried out the Internet-based adaptation of the treatment protocol, with important contributions by RB and AG-P. All authors participated in the review and revision of the manuscript and have approved the final manuscript to be published.

## Funding

This study was financed by the Instituto de Salud Carlos III of the Spanish Ministry of Economy and Competitiveness with the PI10/01083 grant and of the Ministry of Economy and Competitiveness (Plan Nacional I + D + I. PSI2014-54172-R). The project also received funding from the Network for Prevention and Health Promotion in Primary Care (RD12/0005) grant from the Instituto de Salud Carlos III of the Ministry of Economy and Competitiveness (Spain), co-financed with European Union ERDF funds, and the PSI2014-54172-R grant from the Ministry of Economy and Competitiveness (Spain).

The Instituto de Salud Carlos III of the Spanish Ministry of Economy and Competitiveness with the PI10/01083 grant (Eficacia y coste-efectividad de un programa de psicoterapia asistida por ordenador para el tratamiento de la depresión mayor en atención primaria: estudio controlado, randomizado y cualitativo); the Network for Prevention and Health Promotion in primary Care (RD12/0005) grant; the Institute of Health Carlos III (ISCiii) CIBERobn is an initiative of ISCIII; the Government of Aragón; the Department of Innovation, Research; the University and FEDER “Construyendo desde Aragón”.

## Conflict of Interest Statement

The authors declare that the research was conducted in the absence of any commercial or financial relationships that could be construed as a potential conflict of interest.

## Abbreviations

ANOVA, analysis of variance; BDI-II, Beck Depression Inventory-II; CBT, cognitive behavioral therapy; *DSM-IV*, *Diagnostic and Statistical Manual of Mental Disorders, Fourth Edition*; GP, general practitioner; IBIs, Internet-based interventions; ICTs, information and communication technologies; ITT, intention-to-treat; IPDM, individual patient data meta-analysis; M, mean; MINI, Mini International Neuropsychiatric Interview; OASIS, Overall Anxiety Severity and Impairment Scale; ODSIS, Overall Depression Severity and Impairment Scale; PANAS, Positive and Negative Affect Scale; PA, positive affect; NA, negative affect; RCT, randomized control trial; SD, standard deviation.

## Supplementary Material

Data Sheet 1 Canonical correlation analysis.
