# Supplementary material for: Exploring the Relationship Between the Acceptability of an Internet-Based Intervention for Depression in Primary Care and Clinical Outcomes: Secondary Analysis of a Randomized Controlled Trial
Source: Front Psychiatry. 2019 May 10;10:325. doi: 10.3389/fpsyt.2019.00325 (PMC6523778; doi:10.3389/fpsyt.2019.00325)
Supplement: Data Sheet 1 — Canonical correlation analysis. [file DataSheet_1.doc]

Supplementary Material

Table 1. Canonical solution for the relationship between expectations and depression (canonical function 1)

| **Variable** | ***Function 1*** | | |
| --- | --- | --- | --- |
|  | ***Coef*** | ***r*_s_** | $r_{s}^{2}$ **(%)** |
| *Dependent variables*:  ODSIS1  ODSIS2  ODSIS3  ODSIS4  ODSIS5  ODSIS6  ODSIS7  ODSIS8  ODSIS9  ODSIS10  *Predictor variables*:  1. Treatment logic  2. Treatment satisfaction  3. Recommending to others  4. Usefulness for other disorders  5. Usefulness for the patient  6. Unpleasantness* | -.326  .265  -.024  -.128  .373  -.795  .971  -.215  .152  .642  .112  .175  .770  -1.110  -.448  -.512 | .410  .633  .619  .661  .721  .658  .859  .754  .767  .827  -.556  -.462  -.232  -.687  -.616  -.554 | 16.8%  40.1%  38.3%  43.7%  52.0%  43.3%  73.8%  56.8%  58.8%  68.4%  30.9%  21.3%  5.4%  47.2%  37.9%  30.7% |

Notes. *Item 6 is answered in reverse, and so it has been recoded to follow the same scoring criteria as the other 5 items on the scale.

*Coef*: standardized canonical function coefficients. *r_s_*: structure coefficients. Structure coefficients greater than |0.45| are underlined. *r_s_*^2^: squared structure coefficients.

Table 2. Canonical solution for the relationship between expectations and anxiety (canonical function 1)

| **Variable** | ***Function 1*** | | |
| --- | --- | --- | --- |
|  | ***Coef*** | ***r*_s_** | $r_{s}^{2}$ **(%)** |
| *Dependent variables*:  OASIS1  OASIS2  OASIS3  OASIS4  OASIS5  OASIS6  OASIS7  OASIS8  OASIS9  OASIS10  *Predictor variables*:  1. Treatment logic  2. Treatment satisfaction  3. Recommending to others  4. Usefulness for other disorders  5. Usefulness for the patient  6. Unpleasantness* | -.684  .685  -.637  .474  .033  -.107  .261  .778  -.540  .317  -.216  .413  .612  -1.277  .075  -.371 | .094  .503  .356  .609  .558  .593  .708  .720  .476  .681  -.736  -.557  -.455  -.888  -.131  -.606 | 0.9%  25.3%  12.7%  37.1%  31.1%  35.2%  50.1%  51.8%  22.6%  46.4%  54.2%  31.0%  20.7%  78.7%  1.7%  36.7% |

Notes. *Item 6 is answered in reverse, and so it has been recoded to follow the same scoring criteria as the other 5 items on the scale.

*Coef*: standardized canonical function coefficients. *r_s_*: structure coefficients. Structure coefficients greater than |0.45| are underlined. *r_s_*^2^: squared structure coefficients.

Table 3. Canonical solution for the relationship between expectations and negative affect (canonical function 1)

| **Variable** | ***Function 1*** | | |
| --- | --- | --- | --- |
|  | ***Coef*** | ***r*_s_** | $r_{s}^{2}$ **(%)** |
| *Dependent variables*:  PANAS_N1  PANAS_N2  PANAS_N3  PANAS_N4  PANAS_N5  PANAS_N6  PANAS_N7  PANAS_N8  PANAS_N9  PANAS_N10  *Predictor variables*:  1. Treatment logic  2. Treatment satisfaction  3. Recommending to others  4. Usefulness for other disorders  5. Usefulness for the patient  6. Unpleasantness* | -.638  .248  .297  -.078  .884  -.642  .605  -1.482  .832  .328  -.998  .372  1.081  -.405  -.541  .183 | -.041  .288  .425  .308  .450  .245  .300  .142  .526  .461  -.165  .171  .389  -.072  -.603  -.024 | 1.7%  8.3%  18.1%  9.5%  20.2%  6.0%  9.0%  2.0%  27.7%  21.2%  2.7%  2.9%  15.1%  0.5%  36.4%  0.06% |

Notes. *Item 6 is answered in reverse, and so it has been recoded to follow the same scoring criteria as the other 5 items on the scale.

*Coef*: standardized canonical function coefficients. *r_s_*: structure coefficients. Structure coefficients greater than |0.45| are underlined. *r_s_*^2^: squared structure coefficients.

Table 4. Canonical solution for the relationship between expectations and positive affect (canonical function 1)

| **Variable** | ***Function 1*** | | |
| --- | --- | --- | --- |
|  | ***Coef*** | ***r*_s_** | $r_{s}^{2}$ **(%)** |
| *Dependent variables*:  PANAS_P1  PANAS_P2  PANAS_P3  PANAS_P4  PANAS_P5  PANAS_P6  PANAS_P7  PANAS_P8  PANAS_P9  PANAS_P10  *Predictor variables*:  1. Treatment logic  2. Treatment satisfaction  3. Recommending to others  4. Usefulness for other disorders  5. Usefulness for the patient  6. Unpleasantness* | .040  -.510  .589  .042  -.124  .269  -.165  -.486  -.010  -.615  -.475  .670  .055  -.645  -.360  -.390 | -.477  -.643  -.525  -.636  -.724  -.692  -.779  -.900  -.790  -.894  -.816  -.481  -.567  -.761  -.535  -.723 | 22.7%  29.5%  27.6%  40.4%  52.4%  47.9%  60.7%  81.0%  62.4%  79.9%  66.6%  23.1%  32.1%  57.9%  28.6%  52.3% |

Notes. *Item 6 is answered in reverse, and so it has been recoded to follow the same scoring criteria as the other 5 items on the scale.

*Coef*: standardized canonical function coefficients. *r_s_*: structure coefficients. Structure coefficients greater than |0.45| are underlined. *r_s_*^2^: squared structure coefficients.
